# A Case of Inverted Uterus Successfully Treated; Two Cases of the Spontaneous Evolution of the Fœtus; and an Instance of the Cæsarean Operaration Performed by a Woman on Herself

**Published:** 1785

**Authors:** 


### VI. *A Case of inverted Uterus successfully treated; two Cases of the spontaneous Evolution of the Fœtus; and an Instance of the Cæsarean Operation performed by a Woman on herself. Communicated in a Letter from Mr.* Thomas Cawley, *late Surgeon of His Majesty's Military Hospital in Jamaica, to* Robert Adair, *Esq. Inspector General of His Majesty's Military Hospitals, and by him to Dr.* Simmons.

SIR,

I Take the liberty of sending you a case of inverted uterus; two cases of the spontaneous evolution * of the fœtus; and an instance of the Cæsarean operation performed by a woman on herself; which I beg the favour of you to communicate to Dr. Simmons, to be published in the London Medical Journal, if, on examination, they are deemed worthy of a place in that useful collection.

I am, SIR,

With the greatest respect and gratitude,

Your most obedient servant,

THOMAS CAWLEY.

*Chester,*

Sept. 23, 1785.

* See Vol. V. p. 64 and 301.

I shall

I ſhall introduce the caſe of inverted uterus by an extract of a letter from Dr. F. Rigby Brodbelt, dated at Spaniſh Town, in Jamaica, June 20, 1785, and afterwards deſcribe it more particularly, as it appeared to myſelf.

"Mary Aiſlow's caſe was this: — In the "middle of a night in 1783, Mr. Lee deſired "I would go with him to ſee a caſe that he did "not know what to make of. When I got "there and examined it, I was perfectly ſatiſ- "fied it was an inverted uterus, which was "much ſwelled and inflamed, the midwife "having pulled at it with violence, taking "it for the head of a child; for the child and "placenta had been conveyed away before the "midwife came, who would not have been "ſent for, had not the uterus come down. "I endeavoured by the gentleſt means to re- "duce it, but found it impracticable; there- "fore had her bled, gave her an opiate, "threw up emollient clyſters, kept her upon a "cooling regimen, &c. The next day ſhe "was in the ſame ſituation, and the day after "the ſame. Mr. Lee then ſcarified the uterus "in ſeveral places, which had ſo good an ef- "fect as to leſſen its ſize very much, ſo that "it was reduced that night: — ſhe ſtill conti-

"nues

" nues perfectly well. A great many floughs

" came away."

This Mary Aiflow, who is the fubject of Dr. Brodbelt's letter, is a Muftee* girl of Spanifh Town, about twenty-two years of age, and was clandeftinely delivered of a child at the full period of geftation. The child was murdered and concealed, it being intended that this tranfaction fhould be concealed from the world, in order to avoid the difgrace which, in Jamaic, falls rather feverely on a white woman, or one nearly white, who is known to cohabit with a black † man, (though the re-

* A Muftee is the next colour to white, being the offfpring of a white man and Quadroon woman; as a Quadroon is of a white father and Mulatto mother; and a Mulatto of a white father and black mother; or, *vice verfa*, where the father in each of thefe varieties of breed is black; but inftances of the latter are very rare, owing to the ambition of the women to improve the colour in their offspring, and the infamy attending the degradation of it by any long ftrides.

† Thofe of the fame degree of colour frequently contract connections of cohabitation, or even marry; as do alfo brown men with women of a darker colour. Thefe, however, on the woman's fide, are confidered as originating from neceffity rather than choice, and as the complection, if not improved, is not injured—ftand neuter between credit and difhonour.

verfe of fuch cohabitation on a *bonâ fide* agreement, is, among women of colour, not only univerfally common, but efteemed an honourable practice); this was the only apparent caufe, and, confidering the deteftation in which fuch a connexion is held there, the ftrongeft inducement to fo horrid an act.—Marriage, among people of colour, being held ridiculous, illegitimate birth and connexion are not only not fhameful, but of as great repute as lawful defcent or cohabitation among white people.

I accompanied Dr. Brodbelt to fee this patient early on the morning after he was firft called to her affiftance. The tumor was about the fize of a new-born child's head: its whole furface was raw and bloody: at the bottom was an opening which admitted the whole length of a female catheter, and from the mouth of this opening hung a white, twifted, irregular chord, very like the funis umbilicalis. This chord was found to adhere to and line all the cavity from the bottom of the tumor upwards, and, on being untwifted, was nearly fufficient to cover the whole denuded furface of the tumor. This proved that it was the *decidua* defcribed by Dr. Hunter, and the old woman's acknowledgement of violence accounted for its being in this ſtate. A catheter paſſed round the tumor, in the vagina, the uſual depth of that paſſage. All theſe circumſtances, joined to the teſtimony of neighbours reſpecting the woman's pregnancy, were a convincing proof that the tumor was a partial inverſion of the uterus, the lower part having deſcended whilſt the fundus remained up; from which it may be conjectured that the placenta had adhered to the lower part, otherwiſe the fundus uteri would have been pulled down, and the inverſion rendered complete.

In the courſe of this day the whole ſurface of the tumor was in ſuppuration, and, as I attempted to introduce a catheter to draw off the urine, (which was occaſionally neceſſary) a large quantity of very fœtid matter was diſcharged from the vagina. This evening, or the next day, a fever came on, with every appearance, in the tumor, of gangrene from ſtrangulation; for which reaſon it was thought right to ſcarify the uterus, which was accordingly done by Mr. Lee. A great quantity of bloody ſerum iſſued from the inciſions, and the ſwelling immediately diminiſhed, &c. as already related by Dr. Brodbelt. The fever then intermitted, intermitted, and the bark was adminiſtered in large quantities. The applications to the tumor were bark fomentations and poultices. The woman quickly recovered her health and ſtrength.

The remaining caſes are extracts from the letter above quoted.

" I have lately had two curious caſes in mid-

" wifery.—In the firſt I found the arm pre-

" ſenting, the waters totally diſcharged, and

" the uterus ſo contracted about the child as

" to foil me in very many attempts to puſh up

" the arm, and to go up for the feet. The

" woman being very ſtrong, I bled her largely,

" gave her opiates, threw up an emollient

" clyſter every two hours, and injected ℥iv.

" of oil into the vagina every hour. At the

" end of twelve hours violent pains came on,

" and nature delivered the child, which the

" attendants thought was done by me.—The

" ſecond caſe was exactly the ſame, and ter-

" minated as happily in about ſeventeen hours;

" but in this I did not attempt to puſh up the

" arm, and to go up for the feet, as the uterus

" was ſo very much contracted, and the other

" caſe did ſo well;—both the women lived,

" and one of the children."

" Dr. Morton has been in England upwards

" of twelve months; but I can tell you the

" hiſtory of the caſe you wrote about. — A

" negro woman, the property of Mrs. Bland,

" was taken in labour at the Ferry, and not

" being able (as ſhe termed it) to bear the la-

" bour pains, ſhe took a very ſharp knife and

" made a deep inciſion, and extracted the

" child and placenta herſelf;—the inciſion was

" ſo deep as to wound the buttock of the

" child; — after ſhe had done this, ſhe cried

" out for help, and a negro horſe doctor was

" ſent for from Ellis's Crawl, which, you

" know, is about a mile diſtant from the

" Ferry. As ſoon as this knowing doctor

" came, he ſewed up the wound, from one

" end to the other, in the ſame way that we

" ſew up dead bodies, which ſoon produced

" great pain, tenſion, &c. Dr. Morton was

" then ſent for, who ſaw her as ſoon as he

" could. He immediately cut away all the

" ſtitches, and applied ligatures in a proper

" manner, dreſſed her, and did ſuch things

" as he thought proper. The child died the

" next day. Every thing went on in the moſt

" favourable manner with the woman until

" the

" the feventh day; fhe was then feized with
" dyfentery, and died on the ninth day."

